# The role of parity in the relationship between endometriosis and pregnancy outcomes: a systematic review and meta-analysis

**DOI:** 10.1530/RAF-22-0070

**Published:** 2023-03-28

**Authors:** Yorain Sri Ranjan, Nida Ziauddeen, Beth Stuart, Nisreen A Alwan, Ying Cheong

**Affiliations:** 1 Human Development and Health, University of Southampton, Southampton, UK; 2 School of Primary Care, Population Sciences and Medical Education, Faculty of Medicine, University of Southampton, Southampton, UK; 3 NIHR Applied Research Collaboration Wessex, Southampton, UK; 4 Centre for Evaluation and Methods, Wolfson Institute of Population Health, Faculty of Medicine and Dentistry, Queen Mary University of London, London, UK; 5 NIHR Southampton Biomedical Research Centre, University of Southampton and University Hospital Southampton NHS Foundation Trust, Southampton, UK; 6 Complete Fertility, Princess Anne Hospital, Southampton, UK

**Keywords:** endometriosis, pregnancy outcomes, parity, multiparous, primiparous

## Abstract

**Graphical Abstract:**

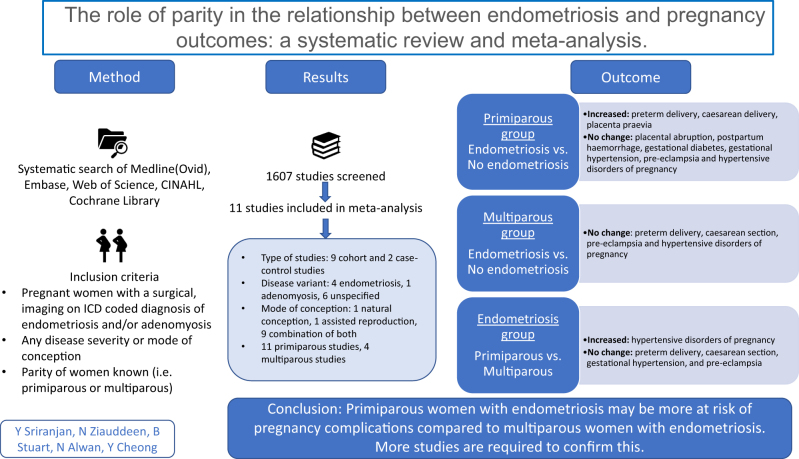

**Abstract:**

Endometriosis is a chronic and debilitating condition which can affect the entire reproductive life course of women with a potentially detrimental effect on pregnancy. Pregnancy (and increasing parity) can affect endometriosis by modulating disease severity and suppressing symptoms. Multiparous women could be less likely to suffer from endometriosis-related pregnancy complications than primiparous women. We aimed to systematically review the evidence examining the role of parity in the relationship between pregnancy outcomes and endometriosis. A systematic search of MEDLINE, EMBASE, CINAHL, Web of Science, and Cochrane Library was performed from inception to May 2022. We searched for experimental and observational studies. Grading of Recommendations, Assessment, Development, and Evaluation was used to assess the quality of evidence with the risk of bias in non-randomised studies of interventions tool incorporated. Eleven studies were included in the meta-analysis. Primiparous women with endometriosis had almost double the risk of hypertensive disorders of pregnancy (OR: 1.99, 95% CI: 1.50–2.63, *P* < 0.001) compared to multiparous women with endometriosis. Primiparous women with endometriosis were at significantly increased risk of preterm delivery, caesarean delivery, and placenta praevia compared to primiparous women without endometriosis. There were no significant differences in outcomes when multiparous women with endometriosis were compared to multiparous women without endometriosis. There is limited evidence to suggest that primiparous women with endometriosis may be at higher risk of adverse pregnancy outcomes compared to multiparous women. The modulatory role of parity in the pathophysiology of endometriosis and its impact on pregnancy outcomes should be investigated.

**Lay summary:**

Endometriosis can adversely affect pregnancy and cause complications that can affect both mother and baby. The severity and symptoms of endometriosis are lessened in pregnancy and with increasing births. Women who have previously given birth could experience fewer pregnancy complications than women giving birth for the first time. We reviewed the literature to compare pregnancy outcomes in women with endometriosis by whether they had given birth before or not. Our review included 11 studies. More women with endometriosis giving birth for the first time had blood pressure disorders in pregnancy than women with endometriosis who had given birth before. First-time mothers with endometriosis tended to have a baby born early, caesarean delivery, and an abnormally located placenta compared to those without endometriosis. This study supports the theory that women with endometriosis in their first pregnancy are at higher risk of complications and may benefit from additional monitoring.

## Introduction

The chronic and debilitating nature of endometriosis, its complex and elusive aetiology coupled with its largely inadequate options for treatment has made endometriosis the focus of much-needed research attention for many years (Higgins *et al.* 2003, Horne *et al.* 2017). The socioeconomic burden of the disease is often underestimated and is predominantly precipitated by the loss of productivity secondary to diminished quality of life in chronic sufferers (Simoens *et al.* 2012). In the United Kingdom, the total annual economic costs of endometriosis-related morbidity are estimated to be ~£8 billion (Simoens *et al.* 2012).

The detrimental effect of endometriosis throughout the entire reproductive life course of a woman is now emerging. The commencement of menarche, which can herald a plethora of deleterious symptoms, can affect the adolescent all the way through adulthood to menopause; the debilitating influence on fecundity can precipitate infertility and the ensuing risks of pursuing assisted reproductive treatments (ARTs). Once pregnant, the negative impact on the course of the pregnancy can affect the mother, the neonate, and potentially have a lifelong effect on the health of the offspring itself; the latter could be explained by the phenomenon of developmental origins of health and disease (Barker 2007). Numerous reviews have highlighted the adverse effects both endometriosis and adenomyosis have on fertility and birth outcomes. Endometriosis increases the risk of spontaneous miscarriage and negatively impacts ART-related outcomes by reducing the oocyte yield and the number of mature oocytes (Horton *et al.* 2019). Furthermore, secondary to impaired folliculogenesis, decreased embryo quality and defective implantation, a reduction in fertilisation and clinical pregnancy rates, and an increased miscarriage rate are often seen in these patients (Carvalho *et al.* 2012, Horton *et al.* 2019). Additionally, endometriosis has been shown to increase the risk of birth-related complications, such as caesarean sections (CS), preterm delivery (PTD), placenta praevia (PP), and placental abruption (PA), and maternal medical disorders such as gestational diabetes (GDM) and hypertensive disorders of pregnancy (HDP) (Farland *et al.* 2019, Horton *et al.* 2019, Razavi *et al.* 2019). These complications are thought to be a direct influence of deferred implantation, progesterone resistance, and altered uterine contractility, resulting in misguided embryo placement, suboptimal placentation, and placental insufficiency (Leone Roberti Maggiore *et al.* 2016). Furthermore, fetal complications such as small for gestational age (SGA), stillbirth, and risks associated with the neonate such as admission to neonatal intensive care unit (NICU) and neonatal death are all increased in mothers with endometriosis (Lalani *et al.* 2018, Horton *et al.* 2019).

It is clear that endometriosis can affect each segment of a woman’s reproductive journey and influence the health of her progeny. Therefore, understanding the natural progression of the disease through important life and reproductive events such as pregnancy and thereby the influence of parity is vital to our knowledge of elucidating disease physiology. The relationship between endometriosis and parity is ambiguous with no definite consensus on how previous pregnancies and parity really influence the disease process and progression. Traditionally, women with endometriosis are advised that becoming pregnant can be a successful strategy in both managing their symptoms and ameliorating disease progression (Leone Roberti Maggiore *et al.* 2016). Historic (and present) observations of regression of endometriomas during pregnancy and lactation and the use of progesterone to create a state of ‘pseudopregnancy’ as a treatment option all support this general belief (Benaglia *et al.* 2013, Leeners *et al.* 2018). Additionally, it is thought that pregnancy and lactation cause hormonal changes, particularly a progesterone-dominant hormonal milieu, which may interfere with the implantation of endometrial lesions (Shafrir *et al.* 2018) and to some extent modulate disease severity. From an epidemiological standpoint, endometriosis in multiparous women tends to be more asymptomatic and less severe (mostly minimal to mild) compared to nulliparous women (Kirshon *et al.* 1989, Sangi-Haghpeykar & Poindexter 1995).

If the underlying disease process is modulated due to the hormonal changes that occur during pregnancy and lactation, the same could be assumed of the pathological processes that govern the development of adverse pregnancy complications due to endometriosis. Assuming this is true, then one could argue the higher the parity (more pregnancies per lifetime), the better the pregnancy outcomes will be due to the improvement of the underlying disease. Similarly, compared to women with endometriosis who are pregnant for the first time (primiparas), we can assume women who have had previous pregnancies and been subjected to its disease ‘modifying effect’ will have better pregnancy outcomes.

Therefore, the aim of this systematic review is to explore the current literature for evidence of studies that examine the role of parity (first pregnancy and subsequent pregnancies) in shaping the reproductive and pregnancy outcomes of women with endometriosis. Where it was appropriate, a meta-analysis for selected outcomes stratified according to parity was performed.

## Materials and methods

### Search strategy

The study was reported in accordance with the Preferred Reporting Items for Systematic Review and Meta-Analysis (PRISMA) guidelines (Moher *et al.* 2009). The protocol for this systematic review was registered on the International Prospective Register for Systematic Reviews (PROSPERO; registration ID: CRD42020173663) and can be accessed at https://www.crd.york.ac.uk/PROSPERO/display_record.php?ID=CRD42020173663.

A systematic search of published studies was performed using the electronic databases MEDLINE, EMBASE, CINAHL, Web of Science, and the Cochrane Library. Studies from database inception to February 2020 with no language restrictions were searched in a systematic manner. Prior to the submission of the review, an updated search was performed in May 2022 to ensure that no newer relevant studies had been published since the last literature search. The keywords include endometriosis, adenomyosis, parity, primiparous, multiparous, reproductive outcomes, obstetric outcomes, and neonatal outcomes. The full search terms are included in the supplementary data (see section on supplementary materials given at the end of this article). Bibliographies and citations of identified articles including review articles (systematic reviews and meta-analyses) were hand-searched and relevant articles were extracted. Ethical approval was not required because data were retrieved from published papers. Patient consent is not applicable.

### Inclusion criteria

All studies which met the inclusion criteria as described later were included. We searched for both experimental and observational studies. Studies which included other co-existing gynaecological pathologies such as fibroids and polycystic ovaries as a main exposure or noted any reference to them were excluded. Conference abstracts where data could be fully extracted were included. Animal studies were excluded.

All studies which included women with a clear recording of their parity status and a diagnosis of adenomyosis or any stage or severity of endometriosis and relevant reproductive, obstetric, and neonatal outcomes (as defined later) were included. Studies where the entire study population comprised either primiparous women only or multiparous women only were included. Furthermore, studies which contained a subgroup analysis of outcomes according to parity were also included (for example, if a study included a subgroup analysis for primiparous women, then this data was included). Where studies have included outcomes data for one subgroup (for example, primiparous women) and values for the total population (which in effect includes both parities), the outcomes data for the remaining subgroup (multiparous women) were calculated through simple subtraction of the two outcome data. Women who conceived through any mode of conception including natural conception (NC), ART, and either were also included as were women who had their endometriosis surgically treated and not treated. Each study also needed to have an appropriate control group consisting of women with no prior diagnosis of endometriosis.

#### Definition of parity

Women either due to give birth to their first child following the diagnosis of endometriosis (primiparous women) or in their subsequent pregnancies where the diagnosis was made any time prior to the index pregnancy (multiparous women) with a clear distinction between the two parity groups were included. For the study to be included in the review, the parity status of all participants needed to be described in the study and ideally also have a breakdown of the outcomes for all participants stratified according to parity. Data on the timing of diagnosis of endometriosis in relation to the pregnancy was also collected where available.

#### Exposure

Endometriosis of any disease severity (mild, moderate, severe, stage 1–4) together with adenomyosis was the exposure of interest. All included studies had the diagnosis of endometriosis made either by confirming the presence of lesions at surgery (with or without histological confirmation), by imaging modality or by International Classification of Disease (ICD)-coded medical records. The diagnosis of adenomyosis was made using either imaging modality or ICD-coded medical records.

#### Outcome measures

Obstetric outcomes measured include delivery by CS, GDM (diabetes diagnosed after 16 weeks of gestation), gestational hypertension (persistently raised blood pressure (≥140/90 mmHg) starting after the 20th week of gestation in an otherwise normotensive woman), pre-eclampsia (PET; gestational hypertension with proteinuria), HDP (includes both PET and gestational hypertension cases), PP (placenta partially or completely covering the internal cervical os during the third trimester), placental abruption (PA; separation of the placenta from its site of implantation before delivery), and post-partum haemorrhage (PPH; blood loss of >500 mL following vaginal delivery or >1000 mL following caesarean).

Neonatal outcomes include PTD (spontaneous birth of an infant between 24 + 0 and 36 + 6 weeks), SGA (birthweight < 10th centile for gestational age), admission to the neonatal unit, and low birth weight (LBW; birthweight < 2500 g at term).

### Selection of studies

Following the search of electronic databases and other sources, the removal of duplicates was performed using EndNote (The EndNote Team 2013). The screening of titles and abstracts for relevance was performed by two independent reviewers (YS and NZ) using Rayyan (Ouzzani *et al.* 2016). All articles were screened by YS and a random sample of 10% was reviewed by NZ. Any discrepancies (*n* = 3) were resolved by a third reviewer (YC). The rate of agreement between the two reviewers at the title and abstract screening phase was 97%. The full texts of all potentially eligible studies were reviewed by YS and those that met the inclusion criteria were included in both the systematic review and meta-analysis.

### Data extraction

Data extraction was performed using a pre-determined data collection form by YS. An independent sample data extraction of studies published between 2005 and 2010 was performed by NZ for comparison (*n* = 4). No discrepancies were identified. Extracted data included study setting and location; study population; parity of participants; study and control groups; reproductive, obstetric, and neonatal outcomes; and information for assessment of the risk of bias. Authors were contacted where missing data on parity was present. Seven authors were contacted and two replied. No further data were gained from these replies. Studies and extracted data were then grouped according to parity as follows. Studies which included only primiparous women or where data on outcomes could be clearly extracted for the primiparous subgroup of the study population were grouped as ‘primiparous studies’. Similarly, studies which included only multiparous women or where data on outcomes could be clearly extracted for the multiparous subgroup were grouped as ‘multiparous studies’.

### Comparative analysis

The intention of our primary analysis was to compare the effect of endometriosis on the main outcomes (reproductive, obstetric, and neonatal) by parity status. This included three comparative groups, namely, primiparous women with endometriosis compared with multiparous women with endometriosis; primiparous women with endometriosis compared with primiparous women without endometriosis; and multiparous women with endometriosis compared with multiparous women without endometriosis. If sufficient studies or data were available for each parity group, subgroup analysis was planned to look at treated vs untreated endometriosis and endometriosis vs adenomyosis.

### Statistical analysis

Meta-analysis was performed using Review Manager version 5.4 (RevMan 5.4; Cochrane Collaboration, Oxford, UK) if two or more studies were available for each outcome and studies were deemed to show sufficient clinical homogeneity as assessed by the reviewers. All data were either directly drawn from the original papers or calculated by Y.S. where appropriate published data was available. All data collected on adverse outcomes were dichotomous and results were presented as Mantel–Haenszel odds ratio (OR) and 95% confidence intervals (CIs). The results calculated as risk differences using RevMan 5.4 were also presented. A random effects model was used to pool the OR data. To assess the statistical heterogeneity of included studies in the meta-analysis, which in turn helped determine the generalisability of study outcomes, the quantity* I^2^* was used (Higgins *et al.* 2003). A *P*-value of less than 0.05 was considered statistically significant. Funnel plots generated by RevMan 5.4 were used to test for publication bias to some degree where there were ten or more studies for each outcome. Sensitivity analysis was performed by either removing adenomyosis only studies, removing studies with multiple pregnancies, or removing outlying data.

### Assessment of study quality

The Grading of Recommendations, Assessment, Development, and Evaluation (GRADE) criteria were used to rate the quality of evidence for each study outcome guided by the GRADE handbook (Schünemann *et al.* 2013) by two reviewers (YS and NZ). Additionally, the risk of bias in non-randomised studies of interventions (ROBINS-I) tool, the recommended tool for assessing the risk of bias in individual non-randomised studies by the Cochrane collaboration (Sterne *et al.* 2022), was used as part of GRADE’s certainty rating process (Schünemann *et al.* 2019) and was performed by YS. Evidence tables were synthesised using the GRADEpro Guideline Development Tool (Software). McMaster University and Evidence Prime, 2022 (available from gradepro.org).

## Results

**Figure 1:**
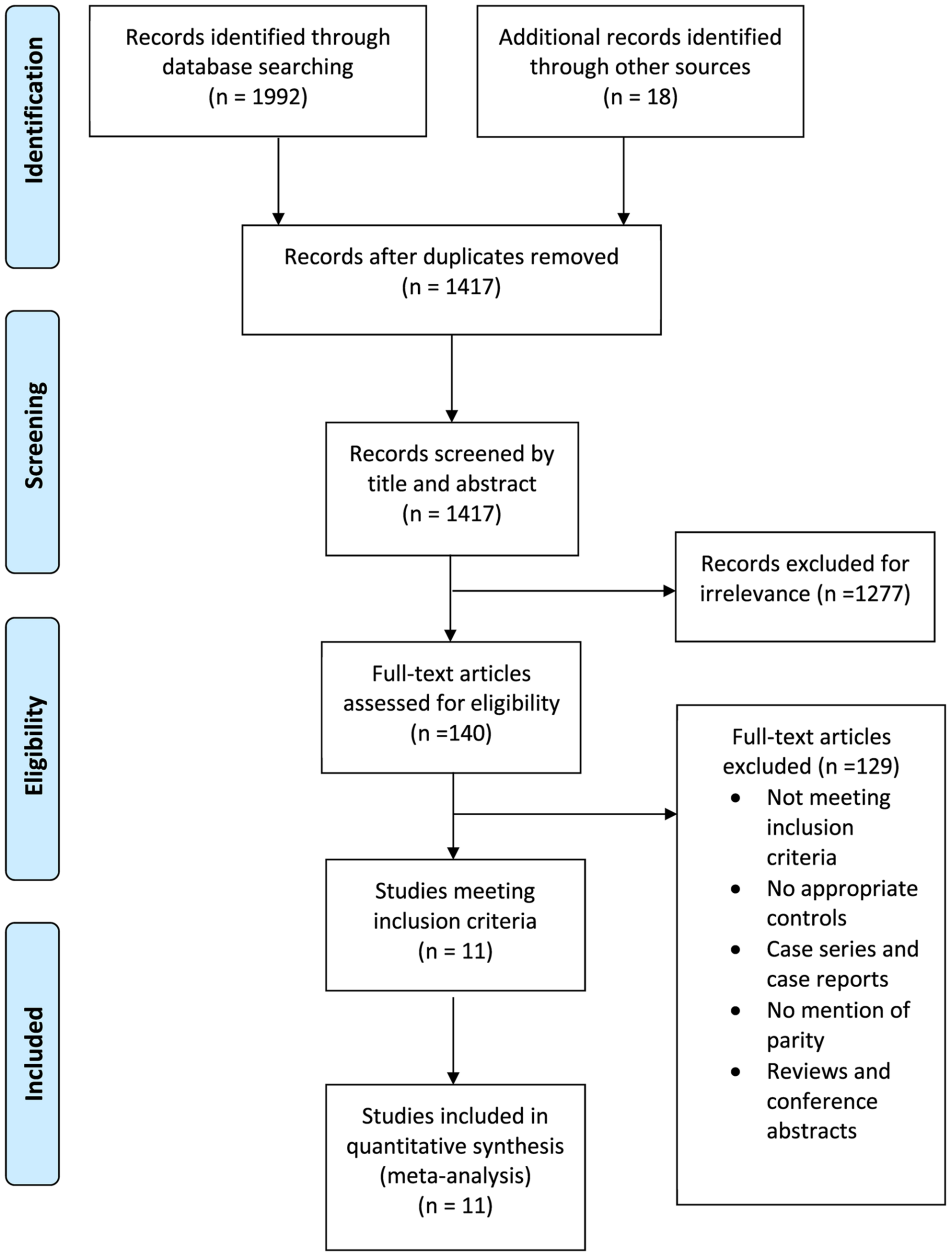
PRISMA flowchart for the study selection process.

A total of 2010 studies were identified through the systematic search, 1992 through the database search and 18 through handsearching of citations and bibliographies of relevant articles. After screening titles and abstracts and removing duplicates and irrelevant papers, 140 full-text articles were assessed for eligibility (Fig. 1). All studies meeting the inclusion criteria (*n* = 11) were included in the quantitative synthesis. No new studies were identified that met the inclusion criteria in the updated search. All study characteristics of studies included in the meta-analysis are presented in Table 1. The results of the assessment of publication bias and sensitivity analysis are summarised in the supplementary data. Studies of relevance yielded by the search consisted only of observational studies (cohort and case-control).

**Table 1:** Characteristics of all included studies in the meta-analysis.

Reference	Location	Study design	Time period	Population	Mode of conception	Stage of endometriosis/subtype	Intervention	Study group	Control group	Outcomes
Primiparous studies										
Conti *et al.* (2015)	Italy	Multicentre, cohort study	2010–2013	Subgroup of primiparous women with singleton pregnancies (*n* = 1550)	NC/ART	All stages (ovarian, mixed ovarian and peritoneal, mixed ovarian and deep, deep)	Not specified	Women with endometriosis diagnosed at surgery by surgical removal of lesions (*n* = 219)	Women with no history of endometriosis (*n* = 1331)	Increased: SGA, GDM, PTD, PPROM, NICU admissions, longer hospital stays; No change: CS, IOL, OVD, Gest HTN, PET, PPH, PROM
Lin *et al.* (2015)	China	Single centre, retrospective cohort study	1995–2013	Primiparous women with singleton pregnancies (*n* = 498)	NC	Not specified	Not specified	Women with endometriosis diagnosed at surgery and confirmed histologically (*n* = 249)	Women with no clinical, USS, or surgical diagnosis of endometriosis (*n* = 249)	Increased: PTD, PP, CS; No change: HDP, PA, FGR, SGA
Saraswat *et al.* (2017)	UK	Multicentre, cohort study using Scottish national database record linkage	1981–2010	Subgroup of primiparous Scottish women with singleton pregnancies (*n* = 6943) and pregnancies that went beyond 24 weeks (*n* = 6536)	NC/ART	Not specified	Not specified	Women with endometriosis diagnosed at surgery or by ICD-coded medical records at enrolment (*n* = 3315) and whose pregnancies went beyond 24 weeks (*n* = 3058)	Women with no recorded diagnosis of endometriosis at enrolment (*n* = 3628) and whose pregnancies went beyond 24 weeks (*n* = 3478)	Increased: Miscarriage, CS, OVD, PP, PPH, uAPH; No change: PTD, HDP, PA, LBW, SB, NND
Berlac *et al.* (2017)	Denmark	Multicentre, cohort study using the Danish national health register and medical birth register	1997–2014	Subgroup of primiparous Danish women with singleton births (*n* = 455,764)	NC/ART	Not specified	Endometriosis diagnosed +/− treated at surgery included but type not specified	Births to women with endometriosis diagnosed from ICD-coded medical records (*n* = 8190)	Births to women with no diagnosis of endometriosis on medical records (*n* = 447,574)	Increased: PET, eclampsia, HELLP, PA, PP, APH, PROM, PTD, SGA, NND, congenital malformations, uterine rupture, MROP, CS in labour/elective; No change: Gest HTN, SB, PPH, 3rd-/4th-degree tears, OVD, low Apgar
Brosens *et al.* (2007)	Belgium	Multicentre, retrospective, case-control study using a hospital database and postal questionnaire	1991–2004	Subgroup of primiparous women with first pregnancy following IVF (*n* = 357)	ART	Not specified	Not specified	Pregnancies in infertile women with surgically diagnosed endometriosis or endometriosis being the reason for referral for IVF with no surgical findings (*n* = 170)	Pregnancies in women referred for IVF for male factor infertility (*n* = 187)	Decreased: PET
Uccella *et al.* (2019)	Italy	Single centre, retrospective, cohort study using a hospital database	2011–2014	Primiparous women (*n* = 1808)	NC/ART	DIE (+/− other) or ovarian endometriosis (+/− peritoneal) or peritoneal endometriosis only	Treated surgically +/− medically but type of surgery not specified	Women with previous histological diagnosis of endometriosis (*n* = 118)	Women with no suspected or confirmed diagnosis of endometriosis (*n* = 1690)	Increased: CS, OVD, PP, HDP; No change: PTD, GDM, IUGR, Blood transfusion, PPH, NICU admissions
Li *et al.* (2017)	China	Single centre, retrospective, cohort study	2011–2013	Primiparous women with singleton pregnancies (*n* = 375)	NC/ART	Stage I–IV, adenomyosis only excluded	Not specified	Women with a pregnancy following previous laparoscopic diagnosis of endometriosis (*n* = 75)	Women pregnant with no history of gynaecological disease (*n* = 300)	Increased: PPH; No change: CS, PTD, HDP, PP, PA, GDM, low Apgar
Yi *et al.* (2020)	Korea	Multicentre, retrospective, cohort study using national databases	2007–2015	Primiparous women (*n* = 1,938,424)	NC/ART	Not specified	Not specified	Women with an ICD-10-coded diagnosis of endometriosis on medical records (*n* = 44,428)	Women with no ICD-10-coded diagnosis of endometriosis (*n* = 1,893,996)	Increased: CS, PPH, PA, PP, PTD, SB, LBW; No change: PET
Hadfield *et al.* (2009)	Australia	Population based longitudinal study of women in New South Wales using database record linkage	2000–2005	Primiparous women with singleton pregnancies (*n* = 208,879)	NC/ART	All stages of endometriosis but adenomyosis only excluded	Not specified	Women with an ICD-10-coded diagnosis of endometriosis on medical records (*n* = 3239)	Women with no ICD-10-coded diagnosis of endometriosis on medical records (*n* = 205,640)	No change: HDP, PET
Mardanian *et al.* (2016)	Iran	Multicentre, cohort study	1993–1997	Infertile primiparous women (*n* = 202)	NC/ART	Not specified	Not specified	Infertile women with endometriosis diagnosed at laparoscopy (*n* = 101)	Infertile women with no endometriosis at laparoscopy (*n* = 101)	No change: Gest HTN, PET
Hashimoto *et al.* (2018)	Japan	Multicentre, retrospective, case-control study using a hospital database	2000–2014	Subgroup of primiparous women with singleton pregnancies (*n* = 252)	NC/ART	Adenomyosis only	No treatment	Women with adenomyosis diagnosed using imaging (*n* = 42)	Women with no adenomyosis on first trimester TVUS (*n* = 210)	Increased: HDP, PET; No change: Gest HTN
Multiparous studies										
Conti *et al.* (2015)	Italy	Multicentre, cohort study	2010–2013	Subgroup of multiparous women with singleton pregnancies (*n* = 689)	NC/ART	All stages (ovarian, mixed ovarian and peritoneal, mixed ovarian and deep, deep)	Not specified	Women with endometriosis diagnosed at surgery by removal of lesions (*n* = 97)	Women with no history of endometriosis (*n* = 592)	Increased: SGA; No change: GDM, PTD, PPROM, NICU admissions, CS, IOL, OVD, Gest HTN, PET, PPH, PROM
Brosens *et al.* (2007)	Belgium	Multicentre, retrospective, case-control study using a hospital database and postal questionnaire	1991–2004	Subgroup of multiparous women referred for IVF (*n* = 162)	ART	Not specified	Not specified	Pregnancies in infertile women with surgically diagnosed endometriosis or endometriosis being the reason for referral for IVF with no surgical findings (*n* = 75)	Pregnancies in women referred for IVF for male factor infertility (*n* = 87)	No change: PET
Hashimoto *et al.* (2018)	Japan	Multicentre, retrospective, case-control study using a hospital database	2000–2014	Subgroup of multiparous women with singleton pregnancies (*n* = 42)	NC/ART	Adenomyosis only	No treatment	Women with adenomyosis diagnosed using imaging (*n* = 7)	Women with no adenomyosis on first trimester TVUS (*n* = 35)	No change: HDP, PET
Saraswat *et al.* (2017)	UK	Multicentre, cohort study using Scottish national database record linkage	1981–2010	Subgroup of multiparous women with singleton pregnancies that went beyond 24 weeks (*n* = 4403)	NC/ART	Not specified	Not specified	Women with endometriosis diagnosed at surgery or by ICD-coded medical records whose pregnancies went beyond 24 weeks (*n* = 1174)	Women with no recorded diagnosis of endometriosis at enrolment whose pregnancies went beyond 24 weeks (*n* = 3229)	Increased: PTD, CS; no change HDP

NC, natural conception; ART, assisted reproductive techniques; DIE, deep infiltrating endometriosis; ICD, international classification of disease; SGA, small for gestational age; GDM, gestational diabetes mellitus; PTB, pre-term birth; PPROM, preterm premature rupture of membranes; NICU, neonatal intensive care unit; NND, neonatal death; CS, Caesarean section; IOL, induction of labour; OVD, operative vaginal delivery; Gest HTN, gestational hypertension; PET, pre-eclampsia; HDP, hypertensive disorders of pregnancy; APH, antepartum haemorrhage; PPH, postpartum haemorrhage; PROM, premature rupture of membrane; PA, placental abruption; PP, placenta praevia; SB, stillbirth; FGR, fetal growth rate; IUGR, intrauterine growth restriction; LBW, low birth weight; HELLP, haemolysis, elevated liver enzymes and low platelets; MROP, manual removal of placenta.

Eleven studies had data on obstetric and neonatal outcomes on primiparous women either as a whole population (*n* = 6) or as a subgroup of the whole population (*n* = 5). These studies were grouped under ‘primiparous studies’ in Table 1. Of these studies, one study solely looked at women with adenomyosis. In terms of mode of conception, nine studies were NC/ART studies, one study only ART and one study only NC. Only two studies specified if the endometriosis was treated prior to pregnancy. Only one study reported obstetric and neonatal outcomes for multiparous women with endometriosis compared to multiparous women without endometriosis (Conti *et al.* 2015). However, using studies reporting outcomes for primiparous women as a subgroup, data for outcomes in multiparous women and their controls were calculated by subtracting the primiparous outcomes data from the entire study population outcomes data (‘multiparous studies’ in Table 1). Data could be obtained by this method for three studies (Brosens *et al.* 2007, Saraswat *et al.* 2017, Hashimoto *et al.* 2018). This group contained one ART study and one adenomyosis study. Data on multiparous outcomes could not be derived by this method for one study (Berlac *et al.* 2017) because the primiparous subgroup of this study was composed of primiparous singletons only; therefore, the subtracted population would include primiparous multiple pregnancies and all multiparous pregnancies.

No studies were found that directly compared obstetric and neonatal outcomes between primiparous women with endometriosis and multiparous women with endometriosis. All outcome data for this comparison were derived from studies which reported primiparous subgroup data and from which multiparous data could be obtained as described earlier (*n* = 4).

### Study and participant characteristics

Of the 11 studies in the meta-analysis, 9 were cohort studies and 2 were case-control studies. In all studies, the diagnosis of endometriosis was either made at surgery (laparoscopy or laparotomy) with or without histological confirmation, at imaging, or based on ICD-coded medical records. The diagnosis of adenomyosis was made at either ultrasound (USS) or magnetic resonance imaging (MRI). Control groups included women with no endometriosis or adenomyosis diagnosed at either surgery, imaging, or based on their ICD-coded medical records (*n* = 9), male factor infertility (*n* = 1), or general infertility (*n* = 1).

In all studies, information relating to the parity of the study population was derived from the baseline characteristics of the studies and outcomes data. Studies where outcomes were not distinct for each parity group were not included.

Four studies looked at all stages of endometriosis and one study investigated women having a sole diagnosis of adenomyosis. Six studies did not specify disease variants or severity. Of those studies that specified some form of treatment for endometriosis (*n* = 2), treatment was surgical with or without a medical component.

When mode of conception was analysed, one study looked at spontaneously conceived pregnancies, one study at ART pregnancies (either intrauterine insemination or *in vitro* fertilisation with or without intracytoplasmic sperm injection), and nine studies a combination of NC and ART pregnancies.

Eight studies adjusted for confounders in their final analysis. These confounders included a diagnosis of infertility, use of ART, maternal age, socio-economic status, year of delivery, gravidity, and gestation at delivery.

### Quality assessment

Individual study outcomes for which a pooled risk estimate could be derived from the meta-analysis (with a minimum of two studies for each outcome) were assessed for risk of bias and quality of evidence using the ROBINS-I tool (Sterne *et al.* 2022) together with the GRADE criteria (Schünemann *et al.* 2013). In primiparous women, the quality of evidence for the outcomes of PTD, CS, and PP was rated as moderate quality; the outcomes of placental abruption, GDM, gestational hypertension, PET, HDP, NICU admissions, and SGA were rated as low quality; and the outcomes of PPH and LBW as very low. The major factors associated with downgrading an outcome for quality were limitations in the study design and execution (risk of bias), inconsistency and imprecision. The factors associated with increasing the quality of evidence were large effect sizes or sample sizes. In multiparous women, all outcomes were rated as low (PTD, CS) and very low (PET, HDP) marked down due to risk of bias and imprecision factors. Similarly, for outcomes comparing primiparous women with multiparous women with endometriosis, quality was rated as low (PTD, CS, HDP) and very low (gestational hypertension, PET). The detailed assessments, together with their explanations are presented in the supplementary pages. Funnel plot analysis of publication bias was not possible due to an inadequate number of studies for each outcome.

### Outcomes

The obstetric and neonatal outcomes from the meta-analysis for the three comparative groups: primiparous women with and without endometriosis, multiparous women with and without endometriosis, and primiparous and multiparous with endometriosis are summarised in Table 2. The results in the table are presented as both ORs and risk differences.

**Table 2:** Summarised findings from the meta-analyses.

Outcomes	Studies (*n*)	Participants (*n*)	OR	95% CI	*P*-values	RD (95% CI)
Primiparous endometriosis vs non-endometriosis						
Obstetric						
Preterm delivery	5	10,767	**1.61**	**1.14–2.26**	**0.006**	0.04 (0.01, 0.07)
Caesarean section delivery	7	2,404,955	**1.63**	**1.52–1.75**	**<0.001**	0.10 (0.08, 0.13)
Placental abruption	5	2,401,597	1.32	0.98–1.77	0.06	0.00 (−0.00, 0.00)
Placenta praevia	6	2,403,405	**3.94**	**2.82–5.51**	**<0.001**	0.02 (0.01, 0.03)
Postpartum haemorrhage	2	462,300	1.25	0.66–2.34	0.50	0.03 (−0.05, 0.12)
Gestational diabetes	3	3733	1.48	0.78–2.83	0.23	0.03 (−0.04, 0.11)
Gestational hypertension	4	457,768	1.07	0.67–1.71	0.77	−0.00 (−0.02, 0.02)
Pre-eclampsia	7	2,605,428	1.18	0.97–1.45	0.10	0.00 (−0.00, 0.01)
Hypertensive disorders of pregnancy	6	218,348	1.32	0.93–1.86	0.12	0.01 (−0.02, 0.03)
Neonatal						
Admission to NICU	2	3358	1.42	0.90–2.24	0.13	0.02 (−0.01, 0.05)
Small for gestational age	3	457,812	1.75	0.87–3.52	0.11	0.02 (−0.00, 0.04)
Low birth weight	2	1,944,960	1.37	0.82–2.29	0.23	0.02 (−0.01, 0.05)
Multiparous endometriosis vs non-endometriosis						
Obstetric						
Preterm delivery	2	5092	1.36	0.97–1.92	0.08	0.02 (0.00, 0.04)
Caesarean section delivery	2	5092	1.49	0.86–2.57	0.15	0.06 (−0.03, 0.16)
Pre-eclampsia	3	893	1.65	0.14–18.84	0.69	−0.01 (−0.04, 0.03)
Hypertensive disorders of pregnancy	3	5134	1.05	0.34–3.24	0.93	−0.01 (−0.04, 0.02)
Primiparous endometriosis vs multiparous endometriosis						
Obstetric						
Preterm delivery	2	4548	1.49	0.73–3.07	0.27	0.04 (−0.04, 0.13)
Caesarean section delivery	2	4548	1.41	0.96–2.08	0.08	0.07 (0.00, 0.13)
Gestational hypertension	2	365	3.29	0.59–18.23	0.17	0.05 (−0.05, 0.14)
Pre-eclampsia	3	610	1.88	0.47–7.54	0.37	0.01 (−0.01, 0.03)
Hypertensive disorders of pregnancy	**3**	**4597**	**1.99**	**1.50–2.63**	**<0.001**	0.04 (0.03, 0.06)

Values in bold indicate statistical significance. OR, odds ratio; RD, risk difference.

#### Primiparous women (endometriosis vs non-endometriosis)

**Figure 2:**
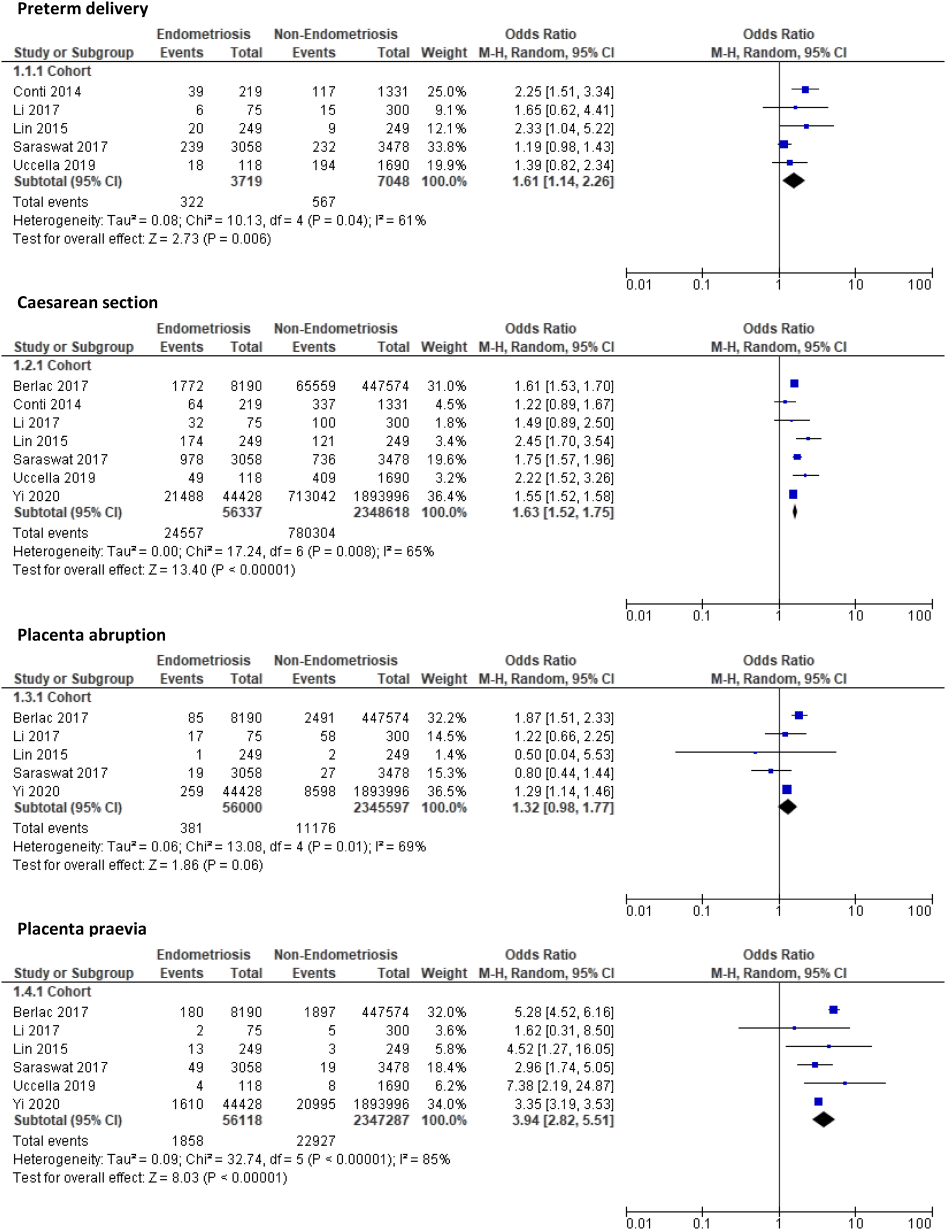

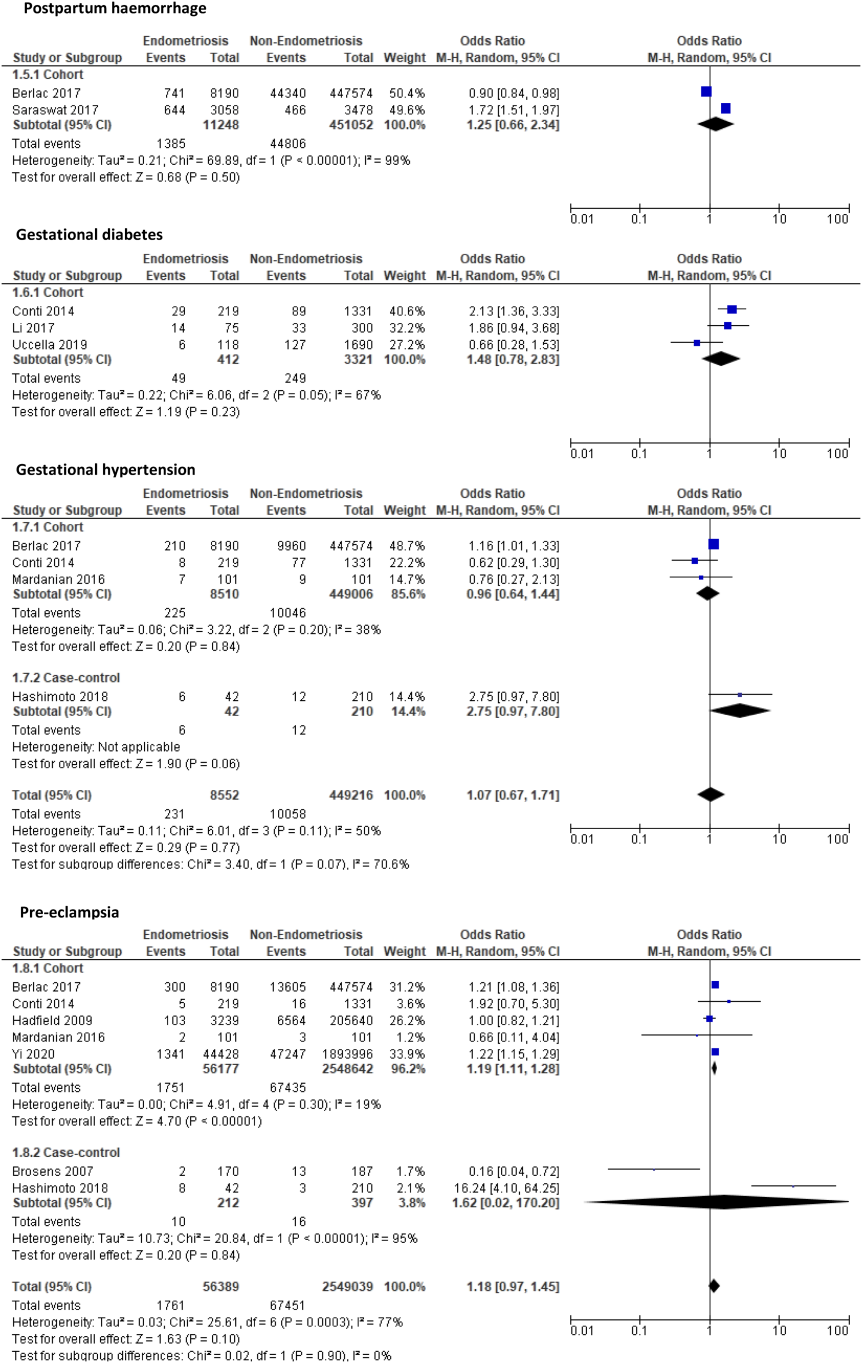

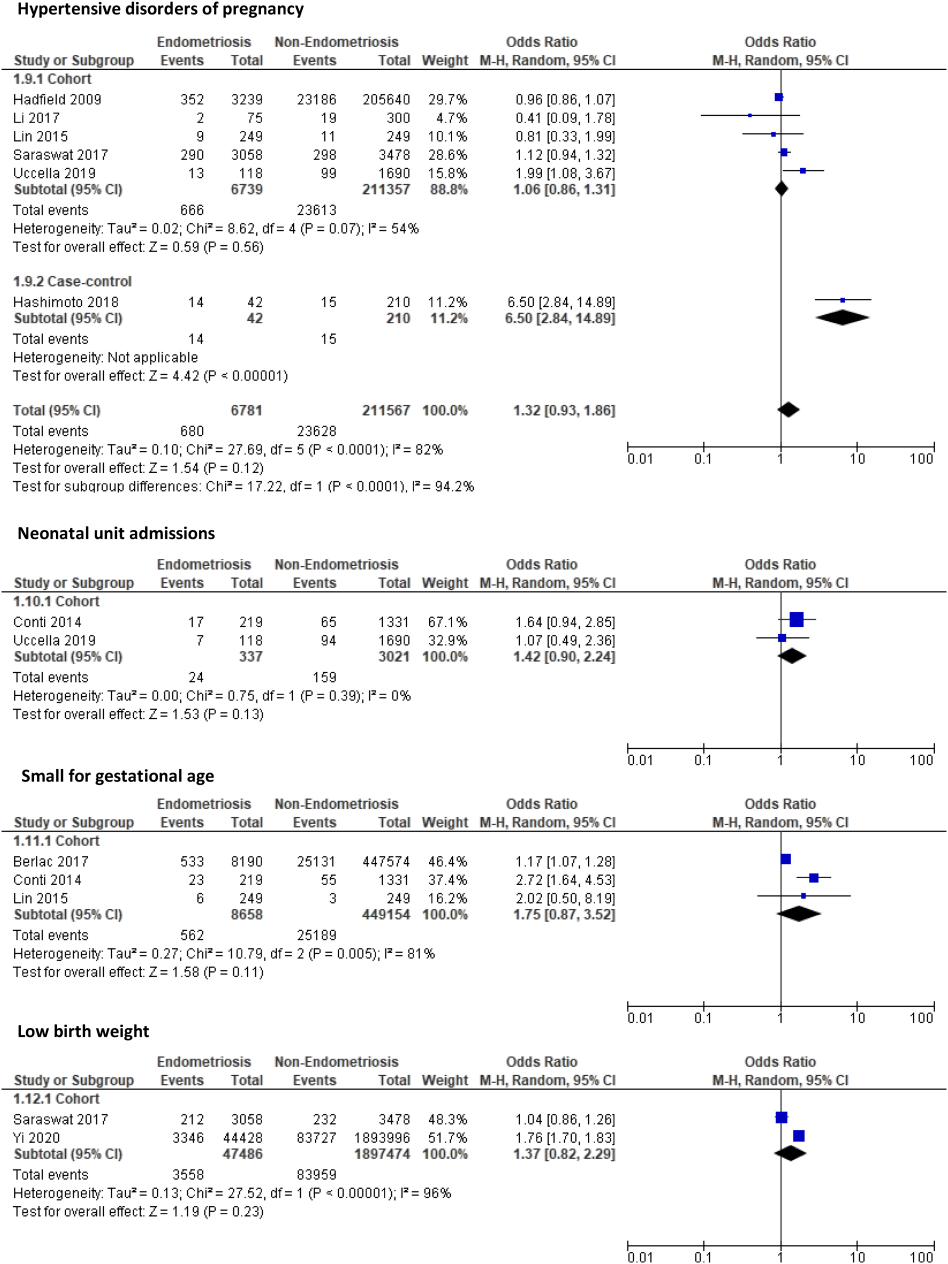
Forest plot comparing pregnancy outcomes in primiparous women with and without endometriosis subgrouped according to study design.

Primiparous women with endometriosis were at significantly increased risk of PTD (OR: 1.61, 95% CI: 1.14–2.26, *P* = 0.006), lower segment CS (OR: 1.63, 95% CI: 1.52–1.75, *P* < 0.001), and PP (OR: 3.94, 95% CI: 2.82–5.51, *P* < 0.001) compared to primiparous women without endometriosis The overall quality of evidence for these outcomes were rated as moderate due to large sample sizes with narrow CIs and, for PP, a large effect size. The risks of PA (OR: 1.32, 95% CI: 0.98–1.77, *P* = 0.06) and PPH (OR: 1.25, 95% CI: 0.66–2.34, *P* = 0.50) were also higher in primiparous women with endometriosis; however, this increase was not significant, and the quality of evidence from the studies assessed was low and very low quality, respectively. The risk of maternal medical disorders such as GDM (OR: 1.48, 95% CI: 0.78–2.83, *P* = 0.23), gestational hypertension (OR: 1.07, 95% CI: 0.67–1.71, *P* = 0.77), PET (OR: 1.18, 95% CI: 0.97–1.45, *P* = 0.10), and HDP (OR: 1.32, 95% CI: 0.93–1.86, *P* = 0.12) were also higher among primiparous women with endometriosis, but this increase was not significant between the two groups and again the quality of evidence was low. Fig. 2 summarises the meta-analyses for each outcome subgrouped according to cohort and case-control studies.

There was no significant change in the risk of admission to the neonatal unit (OR: 1.42, 95% CI: 0.90–2.24, *P* = 0.13), SGA (OR: 1.75, 95% CI: 0.87–3.52, *P* = 0.11), and LBW (OR: 1.37, 95% CI: 0.82–2.29, *P* = 0.23).

#### Multiparous women (endometriosis vs non-endometriosis)

**Figure 3:**
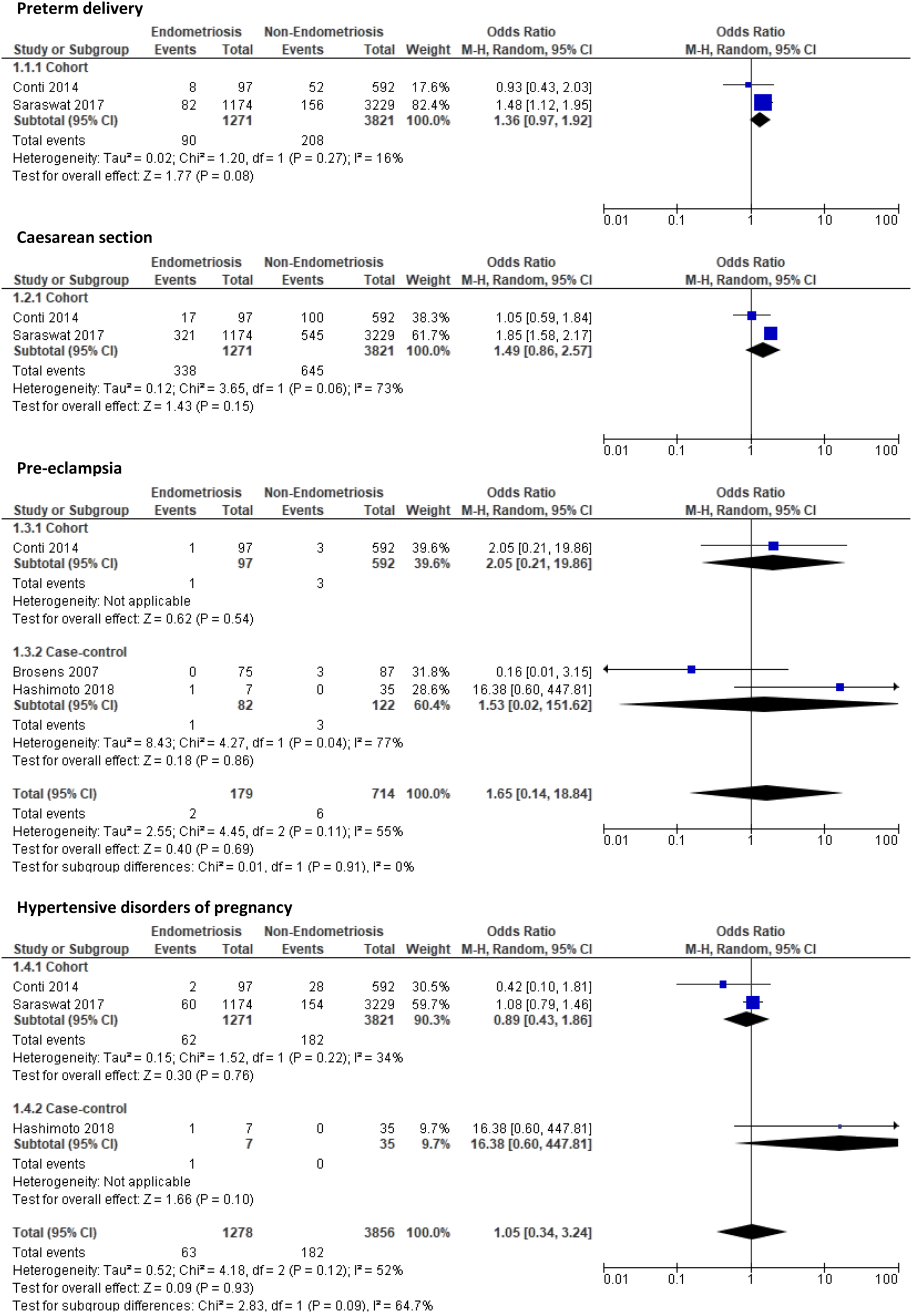
Forest plot comparing pregnancy outcomes in multiparous women with and without endometriosis subgrouped according to study design.

Four study outcomes were compared between multiparous women with endometriosis and those without endometriosis derived from two cohort (Conti *et al.* 2015, Saraswat *et al.* 2017) and two case-control studies (Brosens *et al.* 2007, Hashimoto *et al.* 2018). The obstetrics outcomes of PTD (OR: 1.36, 95% CI: 0.97–1.92, *P* = 0.08), CS (OR: 1.49, 95% CI: 0.86–2.57, *P* = 0.15), PET (OR: 1.65, 95% CI: 0.14–18.84, *P* = 0.69), and HDP (OR: 1.05, 95% CI: 0.34–3.24, *P* = 0.93) showed an increase in multiparous women with endometriosis compared to those without endometriosis, but this increased risk was not significant (Fig. 3). The quality of evidence ranged from low to very low quality for these outcomes marked down due to factors such as the risk of bias in included studies and small sample sizes with wide CIs and/or few events.

#### Primiparous and multiparous women with endometriosis

**Figure 4:**
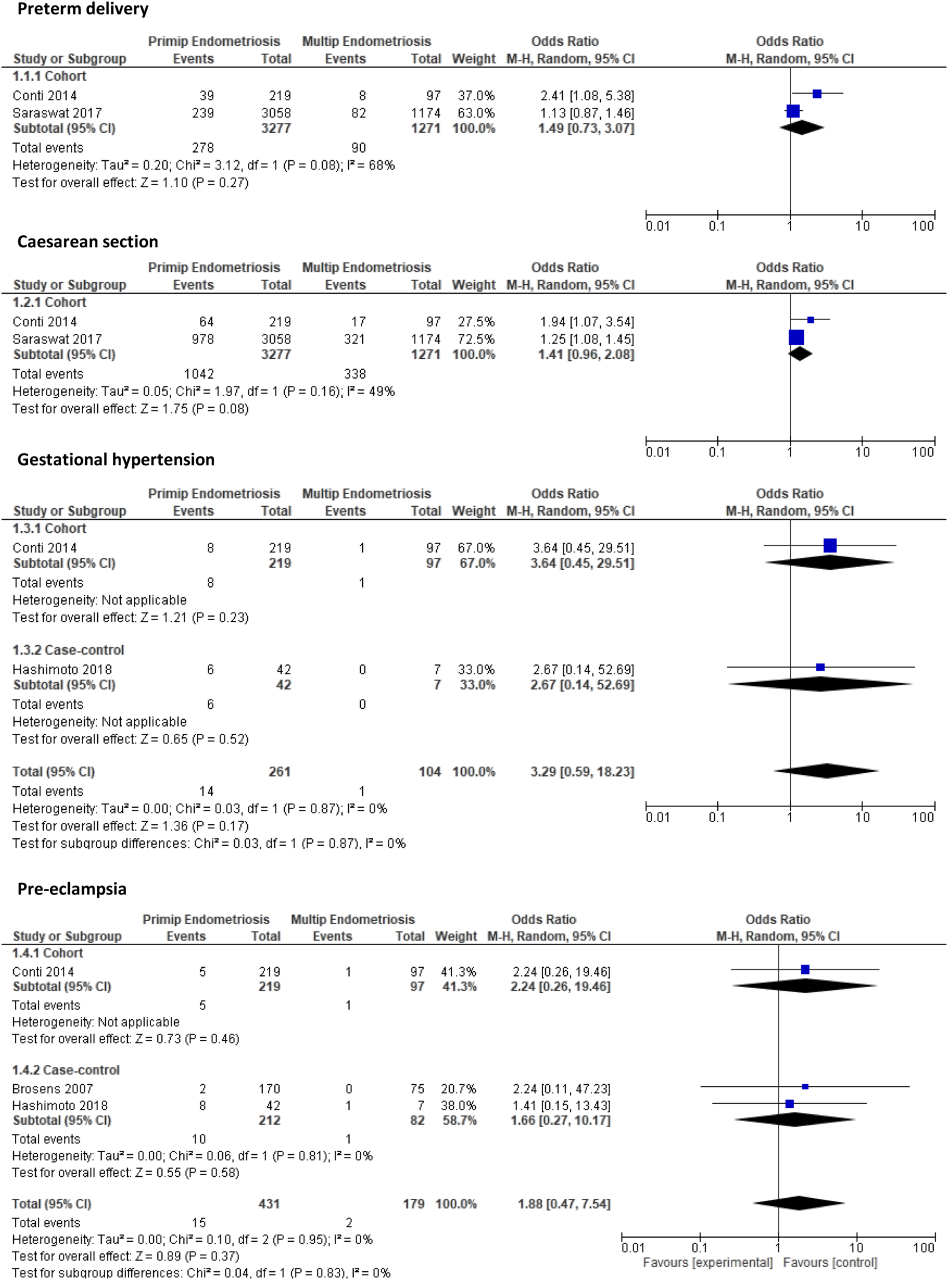

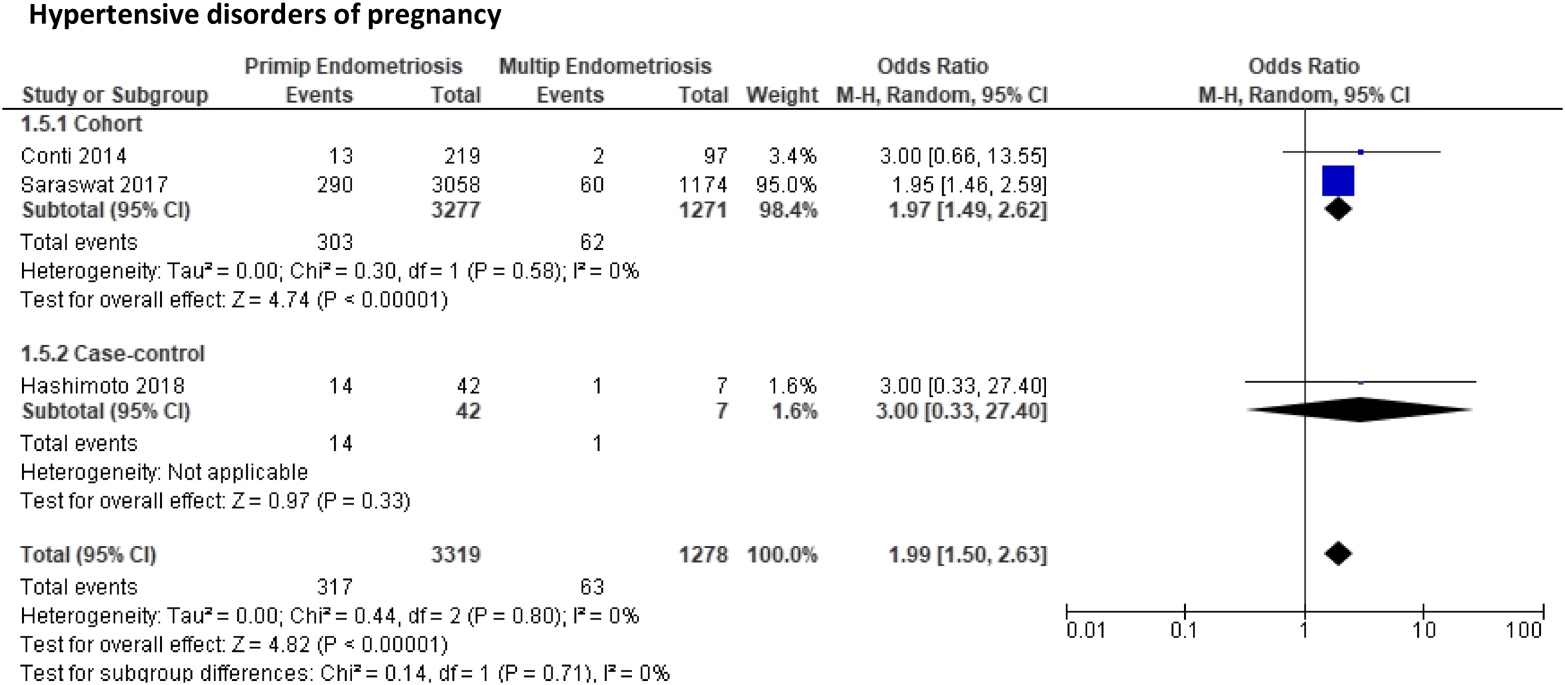
Forest plot comparing pregnancy outcomes in primiparous women with endometriosis and multiparous women with endometriosis subgrouped according to study design.

Five outcomes were compared between primiparous women with endometriosis and multiparous women with endometriosis again derived from two cohort (Conti *et al.* 2015, Saraswat *et al.* 2017) and two case-control studies (Brosens *et al.* 2007, Hashimoto *et al.* 2018). Primiparous women were at an increased risk of PTD (OR: 1.49, 95% CI: 0.73–3.07, *P* = 0.27), CS (OR: 1.41, 95% CI: 0.96–2.08, *P* = 0.08), gestational hypertension (OR: 3.29, 95% CI: 0.59–18.23, *P* = 0.17), and PET (OR: 1.88, 95% CI: 0.47–7.54, *P* = 0.37) compared to multiparous women but these findings were not significant (Fig. 4). In contrast, when the two HDP were combined, primiparous women with endometriosis had a significantly higher risk of HDP compared to multiparous women (OR: 1.99, 95% CI: 1.50–2.63, *P* < 0.001). Again, due to the small sample sizes, few events and wide CI together with high risk of bias in included studies, the quality of evidence was marked down as low and very low quality.

#### Pregnancy outcomes following surgical treatment of endometriosis

Planned subgroup analysis comparing pregnancy outcomes according to treated vs untreated endometriosis for each parity group was not possible due to either lack of studies or general lack of information regarding treatment (example type, proportion treated etc.).

## Discussion

The detrimental effect of endometriosis on reproductive, obstetric, and neonatal outcomes has been well described and extensively studied. Numerous studies, including several meta-analyses, have been undertaken with the aim to consolidate the evidence for this adverse relationship and add to the growing body of evidence of the harmful effect of endometriosis on pregnancy outcomes. Understanding the role of factors such as parity that can potentially influence this relationship is therefore of value as it would, in broad terms, enable risk stratification of pregnant mothers with endometriosis such that a more focused approach to their care can be achieved.

Our meta-analyses found that in women with endometriosis who were giving birth for the first time, the risks of PTD, lower segment CS, and PP were significantly increased compared to women without endometriosis, with the quality of the evidence for each outcome rated a moderate quality using the GRADE criteria ((Schünemann *et al.* 2013).

This contrasts with findings in multiparous women, where none of the outcomes compared, namely, PTD, CS, PET, and HDP showed a significant difference between those with and without endometriosis. This is an interesting observation as this would add weight to our hypothesis that multiparous women, unlike their primiparous counterparts, do not show a tendency to have an increased risk from adverse obstetric outcomes despite having a diagnosis of endometriosis. It is difficult to know if this is a true protective effect rendered by increased parity or an artefact caused by the dearth of study data on multiparous women to show a true effect, with subsequent low to very low-quality evidence.

When primiparous women with endometriosis were compared with multiparous women with endometriosis, neither the obstetric outcomes of PTD and CS nor the outcomes for PET and gestational hypertension showed a significant difference between the two groups. However, it is still noteworthy that, despite these differences not being significant, the tendency was for primiparous women to have an increased risk overall compared to multiparous women. When the two HDP were combined (gestational hypertension and PET), the difference was significant, with primiparous women having almost double the odds of the outcome compared to multiparous women. These findings should perhaps be interpreted with some degree of caution for two reasons. First, first pregnancy is a risk factor for the development of gestational hypertension (BMJ Best Practice 2018) and PET (NICE 2019) regardless of the presence of endometriosis and secondly, the meta-analyses for these outcomes are based on a limited number of smaller studies with few events as most of the multiparous outcome data were derived from studies where suitable published data were available. Therefore, the quality of evidence for these outcomes is low to very low. There were no studies that directly reported comparative outcomes for these groups.

Sensitivity analysis was carried out by removing studies which included multiple pregnancies, had outlying results, and studies investigating only adenomyosis. The overall findings were robust to these potential influential factors.

The traditional line of thinking is that pregnancy renders a positive effect on endometriosis and its symptoms (Leone Roberti Maggiore *et al.* 2016). It is not uncommon for clinicians to advice women that becoming pregnant might give some respite from their debilitating symptoms and might even halt disease progression. The risk of endometriosis declining with parity and the occurrence of symptoms with menarche (childbearing ages) and regression with menopause coupled with some early studies showing the regression of lesions during pregnancy adds to this argument (Bulun 2009). This is the rationale for the initiation of a ‘pseudopregnancy’ or ‘chronic anovulation’ state with long-term progesterone for the suppression and treatment of symptoms (Olive & Pritts 2001). A pregnant mouse model of endometriosis has demonstrated that despite an increase in the size of lesions, cellular proliferation within the lesions decreased and apoptosis increased with an increase in leukocyte infiltration and necrosis (Bilotas *et al.* 2015). In a study of rats with surgically induced endometriosis, significant regression in ectopic implants was noted during lactation (Barragan *et al.* 1992). The ensuing anovulation and amenorrhoea may not only ameliorate the anatomical distortions that result from bleeding endometriotic lesions but also negate the negative hormonal, inflammatory, and angiogenic response of the disease (Leone Roberti Maggiore *et al.* 2016). All this evidence points to a molecular and cellular level modification of disease during pregnancy which can potentially have longstanding benefits, not only in reversing the adverse effects on pregnancy outcomes but also in symptom management and disease progression following birth.

Despite these robust arguments, the evidence for this recommendation appears controversial and studies looking into the longitudinal manifestation of endometriosis during and after pregnancy is scarce and of medium to low quality (Leeners *et al.* 2018). Therefore, drawing meaningful conclusions on the true impact of pregnancy and the ensuing change in parity status on endometriosis is challenging. Also, it is difficult to know whether the changes to the disease observed during pregnancy are transient and limited to the pregnancy and ensuing period of lactation or more long-term such that it can impact future pregnancies and their outcomes, that is, higher the parity, the better the pregnancy-related outcomes. Furthermore, there is no data looking at whether it is the state of being pregnant, even for a brief period as occurs with miscarriages, or pregnancy that is completed to term that should be considered long enough to have a beneficial effect on endometriosis. There are currently no studies evaluating reproductive, obstetrics or neonatal outcomes solely in multiparous women nor any studies looking at the inter-pregnancy variations in outcomes. Indeed when dealing with studies on outcomes for multiparous women, another confounding influence to bear in mind is, not only the timing of endometriosis diagnosis in relation to pregnancy but perhaps also the timing of onset* of* symptoms*,* as it is now well known that there is a considerable lag in the establishment of the former from the onset of the latter (Ghai *et al.* 2020).

The increasing consensus on the negative impact of endometriosis on reproductive, obstetric, and neonatal outcomes regardless of disease severity, mode of conception, and prior treatment necessitates a paradigm shift in the way antenatal patients and those seeking preconceptual advice are counselled. The present study was aimed at risk stratifying this already high-risk group according to parity so that a more targeted approach could be used in delivering antenatal and intrapartum care. Despite the limited number of studies actively comparing pregnancy outcomes by parity in women with endometriosis, there is some evidence to suggest that primiparous women may be more at risk compared to multiparous women; therefore, it would be prudent to be extra vigilant when caring for these women antenatally. This improved surveillance can be in the form of appropriate counselling of risks, regular check of health parameters for the development of maternal medical disorders, and obstetrician-led antenatal care to ensure best outcomes for these women. The present study clearly highlights an important area where future research should focus on.

### Strengths and limitations

This is the first time to our knowledge a systematic review has been undertaken to study the role of parity in the relationship between endometriosis and pregnancy outcomes. No studies have been found in the literature to address this directly to date.

The process of study selection by title and abstract and data extraction using a standardised data collection form was performed by two independent reviewers with minimal discrepancy. Despite the second reviewer performing the selection and extraction for a random sample of studies, the process allowed for study selection bias to be reduced and reinforcement of the method used by the first reviewer. Our review also employed an extensive search strategy incorporating a comprehensive list of obstetric and neonatal outcomes and terminology for parity. GRADE is a transparent and reproducible framework that is used to rate the quality of evidence of studies (Siemieniuk & Guyatt, n.d.). Two independent reviewers graded the certainty of outcomes. As outcomes are all derived from observational data, grading was commenced at ‘low’ quality and was moved up or down the certainty scale depending on the grading domains (Schünemann *et al.* 2013). In all comparative groups, the majority of outcomes were graded as low or very low quality, mainly marked down due to a combination of serious risk of bias (as assessed using the ROBIN-I tool), inconsistency and/or imprecision factor.

In terms of limitations, one of the main drawbacks of this review is the dearth of studies directly looking at endometriosis-related pregnancy outcomes in multiparous women and the complete lack of studies directly comparing pregnancy outcomes between the two parity groups in women with endometriosis. This was mitigated to some extent by the ability to calculate outcomes for multiparous women from suitable published data. However, this was still only possible for a handful of studies. This is reflected in the meta-analyses data where the number of studies for each outcome in multiparous women was considerably lower than the numbers for the primiparous analysis.

The inevitable presence of clinical and methodological heterogeneity and variability in study quality when dealing with multiple observational studies in a systematic review is a known limitation. The differences in study design (cohort vs case-control), study population and control groups (which include infertile women, male factor infertility, and general population), the variability in outcomes (in the way they are defined or measured), mode of conception, disease variant, and risk of bias all contribute to considerable statistical heterogeneity. In order to mitigate this to some extent and ensure studies included in the meta-analysis are sufficiently homogenous in terms of intervention and outcomes, the following steps were undertaken; to improve diagnostic accuracy and uniformity we only included studies that either had endometriosis and/or adenomyosis diagnosed at surgery (gold standard), at imaging, or by ICD-coded medical records. This aimed to eliminate participant respondent bias that would have arisen if data were gathered using questionnaires or patient interviews. The use of the latter two methods can still introduce some degree of error to the diagnosis of endometriosis. Imaging methods such as ultrasound scans are highly user dependent and mostly used for the evaluation of endometriomas (Moore *et al.* 2002); therefore, milder forms of the disease could still be missed. Similarly, medical diagnosis can also be coded inaccurately or subjectively on ICD records although the risk of this is likely low. Furthermore, we only included studies which measured or defined outcomes according to the definitions in our inclusion criteria and specified a priori, thereby ensuring consistency.

Lastly, it is recognised that nulliparity is a risk factor for certain adverse pregnancy outcomes; however, the relationship between parity and adverse pregnancy outcomes is controversial and one that is still being debated. Nulliparity has been linked to a range of obstetric and neonatal complications compared to multiparity (Miranda *et al.* 2011, Chauhan *et al.* 2020). These complications include risks such as PET, PTB, LBW, and SGA among others (Luo *et al.* 2007, Shah 2010, Lin *et al.* 2021). However, not all studies corroborate these findings. A large cross-sectional study conducted in Australia during the period 1992–1997 classified women into three groups according to parity: nulliparous, low multiparous, and grand multiparous (parity 4–8). The cohort included 510,989 singleton births and concluded that compared to low multiparous women, nulliparous, and grand multiparous women had a higher risk of obstetric complications and neonatal morbidity despite adjusting for a range of confounders, suggesting a ‘U’-shaped association between parity and pregnancy outcomes (Bai *et al.* 2002). Several large cohort studies suggest an increased risk in multiparous mothers compared to nulliparous ones. A Canadian study of 123,941 singleton births concluded multiparity is associated with a higher risk of placental bleeding disorders such as PP and abruption (Ananth *et al.* 1996). Another recent study of 133,926 births in China concluded Rubella seronegative multiparous mothers are at an increased risk of PET and perinatal loss (Lao *et al.* 2022). Furthermore, several studies report on higher risks of malpresentation, placenta previa, macrosomia, and low Apgar scores in grand multiparas (Al-Farsi *et al.* 2012, Mgaya *et al.* 2013, Al-Shaikh *et al.* 2017). The current review investigated the effect of parity and endometriosis across a wide range of adverse pregnancy outcomes; however, due to insufficient data, was unable to subgroup by the level of multiparity.

### Implications for future research

It is clear from this review that there is a need for more homogenous, well-designed, and longitudinal studies to address the role of parity on pregnancy outcomes in women with endometriosis in order to make more meaningful inferences. Only one study has addressed this to some extent at present (Conti *et al.* 2015). Ideally, these studies should be designed in such a way that they are able to not only address the contribution of parity on pregnancy outcomes but also allow a better understanding of the contribution of pregnancy and the ensuing postpartum period on the natural progression of the disease as an entirety. Furthermore, a longitudinal design would enable us to glean the effect of endometriosis on successive pregnancies, especially, if any, the inter-pregnancy variations of the disease and how it might affect future pregnancy outcomes. Additionally, an experimental design examining pregnancy outcomes in primiparous women with endometriosis and no other risk factors who are either offered obstetrician-led care with increased surveillance or standard care would be useful in assessing the benefits of streamlined care for this group.

## Conclusion

In conclusion, the findings from our study indicate that primiparous women with endometriosis may be at increased risk of HDP compared with multiparous women with endometriosis. Moreover, primiparous women with endometriosis may also be at increased risk of certain pregnancy outcomes (PTD, CS, and PP) compared to those without the disease. Multiparous women with endometriosis do not appear to be at an increased risk for any of the outcomes compared to their control non-endometriosis counterparts, possibly attributed to the disease-modifying effect of previous pregnancies.

These results are in favour of the hypothesis that primiparous women are at increased risk of adverse pregnancy outcomes to some extent compared to multiparous women with endometriosis. However, it must be highlighted again that the number of studies comparing outcomes in the latter group is limited in the current review and of low to very low quality. The mechanism by which increased parity renders a protective effect from adverse pregnancy outcomes remains to be elucidated. To obtain further insight into the role of parity and previous pregnancies on reproductive outcomes in women with endometriosis, so that clinicians can take cognizance of this fact when counselling pregnant mothers with the condition, future high-quality, well-designed studies aimed at understanding the contribution of this important factor is required.

## Supplementary Material

Supplementary Material

## Declaration of interest

YC is an associate editor for *Reproduction and Fertility*, however, was not involved in the editorial review process. None of the other authors have any conflicts of interest to declare.

## Funding

This study did not receive any specific grant from any funding agency in the public, commercial, or not-for-profit sector.

## Author contribution statement

YC and NAA conceived the study. YS drafted the protocol, developed the search strategy, screened the studies, extracted, and analysed the data and drafted the manuscript. NZ also screened a sample of studies and performed a sample data extraction. YC, NAA, and NZ provided supervisory, conceptual, and analytical support. BS provided statistical advice. All authors edited and agreed the final version of the manuscript.
