# Supplementary material for: The role of parity in the relationship between endometriosis and pregnancy outcomes: a systematic review and meta-analysis
Source: Reprod Fertil. 2023 Mar 28;4(1):e220070. doi: 10.1530/RAF-22-0070 (PMC10083661; doi:10.1530/RAF-22-0070)
Supplement: Supplementary Material [file supplementary_material.pdf]

The role of parity in the relationship between endometriosis and pregnancy outcomes.

Yorain Sri Ranjan, Nida Ziauddeen, Beth Stuart, Nisreen Alwan, Ying Cheong

Supplementary data 1: Full database search terms (free text and Medical Subject Headings [MeSH])

Disease:
Adenomyosis
Endometrio* (covers the following)

Endometriosis
Endometrioma/ ovarian endometriosis
Deep infiltrating endometriosis (DIE)
Mild, Moderate, Stage 1, Stage 2 AND endometriosis
Moderate, Severe, Stage 3, Stage 4 AND endometriosis

Outcome: AND
Fertili*(covers Fertilization rate, fertilisation rate) (MeSH - fertilisation in vivo), implantation rate (MeSH h-embryo implantation), clinical pregnancy rate (MeSH - pregnancy rate), pregnancy rate, cycle cancellation, ovarian response, preterm, prematur*, small for gestational age, growth restriction, pregnancy AND hypertension or gestational AND hypertension (MeSH - pregnancy induced hypertension), pre-eclampsia (MeSH), obstetric cholestasis (intrahepatic-cholestasis and pregnancy complications), caesarean, haemorrhage, placenta praevia, placental abruption (MeSH - with pregnancy complication), miscarriage, livebirth rate (MeSH -livebirth), mid-trimester loss, obstetric outcome, neonatal outcome, pregnancy outcome, perinatal outcome, reproductive outcome, fertility outcome (MeSH - female infertility)

Patient groups: AND
Primip*, multip, multipar*, nullip*, parous, parity (MeSH - parity, first pregnancy, *gravida

Treatment: OR
Treatment AND endometrio*/ AND adenomyosis (open or laparoscopic)
Surgery AND endometrio*/ AND adenomyosis
Excision AND endometrio* AND adenomyosis

The role of parity in the relationship between endometriosis and pregnancy outcomes.

Yorain Sri Ranjan, Nida Ziauddeen, Beth Stuart, Nisreen Alwan, Ying Cheong

Supplementary data 2: Assessment of publication bias through funnel plot analysis

Parity	Outcome	Publication Bias
Primiparous women with endometriosis vs. primiparous women without endometriosis	PTD	Unable to interpret as too few studies
	CS	Unable to interpret as too few studies
	PA	Unable to interpret as too few studies
	PP	Unable to interpret as too few studies
	PPH	Unable to interpret as too few studies
	GDM	Unable to interpret as too few studies
	Gest HTN	Unable to interpret as too few studies
	PET	Unable to interpret as too few studies
	PIH	Unable to interpret as too few studies
	NNU	Unable to interpret as too few studies
	SGA	Unable to interpret as too few studies
	LBW	Unable to interpret as too few studies
Multiparous women with endometriosis vs. multiparous women without endometriosis	PTD	Unable to interpret as too few studies
	CS	Unable to interpret as too few studies
	Gest HTN	Unable to interpret as too few studies
	PET	Unable to interpret as too few studies
	PIH	Unable to interpret as too few studies
Primiparous women with endometriosis vs. multiparous women with endometriosis	PTD	Unable to interpret as too few studies
	CS	Unable to interpret as too few studies
	Gest HTN	Unable to interpret as too few studies
	PET	Unable to interpret as too few studies
	PIH	Unable to interpret as too few studies

The role of parity in the relationship between endometriosis and pregnancy outcomes.

Yorain Sri Ranjan, Nida Ziauddeen, Beth Stuart, Nisreen Alwan, Ying Cheong

Supplementary data 3: Sensitivity analysis

Parity	Outcome	Sensitivity Analysis
Primiparous women with endometriosis vs. primiparous women without endometriosis	PTD	Robust
	CS	Robust
	PA	Robust
	PP	Robust
	PPH	None applied
	GDM	Interpret with caution
	Gest HTN	Robust
	PET	Robust
	PIH	Robust
	NNU	Robust
	SGA	None applied
	LBW	Robust
Multiparous women with endometriosis vs. multiparous women without endometriosis	PTD	Robust
	CS	Robust
	Gest HTN	None applied
	PET	Robust
	PIH	Robust
Primiparous women with endometriosis vs. multiparous women with endometriosis	PTD	Robust
	CS	Robust
	Gest HTN	None applied
	PET	Robust
	PIH	Robust

The role of parity in the relationship between endometriosis and pregnancy outcomes.

Yorain Sri Ranjan, Nida Ziauddeen, Beth Stuart, Nisreen Alwan, Ying Cheong

Supplementary data 4.1: GRADE summary of findings table for obstetric and neonatal outcomes in primiparous women *(endometriosis vs. non-endometriosis)*.

	Certainty assessment							№ of patients		Effect	
Outcome	№ of studies	Study design	Risk of bias (ROBINS-I)	Inconsistency	Indirectness	Imprecision	Other considerations	Endometriosis	Non-Endometriosis	Relative (95% CI)	Certainty
Preterm Delivery	5	observational studies[a]	serious[b]	not serious	not serious	not serious	none[c]	322/3719 (8.7%)	567/7048 (8.0%)	OR 1.61 (1.14 to 2.26)	⊕⊕⊕◯ Moderate
Caesarean Section Delivery	7	observational studies[a]	serious[b]	not serious	not serious	not serious	none[c]	24557/56337 (43.6%)	780304/2348618 (33.2%)	OR 1.68 (1.47 to 1.93)	⊕⊕⊕◯ Moderate
Placental Abruption	5	observational studies[a]	serious[b]	not serious	not serious	serious[d]	none[c]	381/56000 (0.7%)	11176/2345597 (0.5%)	OR 1.32 (0.98 to 1.77)	⊕⊕◯◯ Low
Placenta Praevia	6	observational studies[a]	serious[b]	not serious	not serious	serious[e]	strong association[c,f]	1858/56118 (3.3%)	22927/2347287 (1.0%)	OR 3.94 (2.82 to 5.51)	⊕⊕⊕◯ Moderate
Postpartum Haemorrhage	2	observational studies[a]	serious[b]	serious[g]	not serious	serious[d]	none[c]	1385/11248 (12.3%)	44806/451052 (9.9%)	OR 1.25 (0.66 to 2.34)	⊕◯◯◯ Very low
Gestational Diabetes	3	observational studies[a]	serious[b]	not serious	not serious	serious[d]	none[c]	49/412 (11.9%)	249/3321 (7.5%)	OR 1.48 (0.78 to 2.83)	⊕⊕◯◯ Low

The role of parity in the relationship between endometriosis and pregnancy outcomes.

Yorain Sri Ranjan, Nida Ziauddeen, Beth Stuart, Nisreen Alwan, Ying Cheong

	Certainty assessment							№ of patients		Effect	Certainty
Outcome	№ of studies	Study design	Risk of bias (ROBINS-I)	Inconsistency	Indirectness	Imprecision	Other considerations	Endometriosis	Non-Endometriosis	Relative (95% CI)	
Gestational Hypertension	4	observational studies[a]	serious[b]	not serious	not serious	serious[d]	none[c]	231/8552 (2.7%)	10058/449216 (2.2%)	OR 1.07 (0.67 to 1.71)	⊕⊕◯◯ Low
Pre-eclampsia	7	observational studies[a]	serious[b]	not serious	not serious	serious[d]	none[c]	1761/56389 (3.1%)	67451/2549039 (2.6%)	OR 1.18 (0.97 to 1.45)	⊕⊕◯◯ Low
Hypertensive disorders of pregnancy	6	observational studies[a]	serious[b]	not serious	not serious	serious[d]	none[c]	680/6781 (10.0%)	23628/211567 (11.2%)	OR 1.32 (0.93 to 1.86)	⊕⊕◯◯ Low
Neonatal Admissions	2	observational studies[a]	serious[b]	not serious	not serious	serious[d]	none[c]	24/337 (7.1%)	159/3021 (5.3%)	OR 1.42 (0.90 to 2.24)	⊕⊕◯◯ Low
Small for Gestational Age	3	observational studies[a]	serious[b]	not serious	not serious	serious[d]	none[c]	562/8658 (6.5%)	25189/449154 (5.6%)	OR 1.75 (0.87 to 3.52)	⊕⊕◯◯ Low
Low Birth Weight	2	observational studies[a]	serious[b]	serious[g]	not serious	serious[d]	none[c]	3558/47486 (7.5%)	83959/1897474 (4.4%)	OR 1.37 (0.82 to 2.29)	⊕◯◯◯ Very low

CI: confidence interval; **OR:** odds ratio. **Explanations: a**. Includes both cohort and case-control studies; **b.** Risk of bias due to following; unrepresentativeness of either cases/exposed or controls/unexposed and/or not adjusting for confounders; **c.** There is too few studies to accurately detect funnel plot asymmetry. (<10). Therefore, the possibility of publication bias is not excluded but it is not considered sufficient to downgrade the quality of evidence; **d.** Downgraded one level as even though a large sample size is present (optimal information size criteria is met), the 95% CI overlaps no effect and is wide; **e.** Downgraded one level due to wide 95% CIs; **f.** Effect size large (OR >2); **g.** Downgraded one level as statistically significant, unexplained

heterogeneity of results detected due to large variation of effect in studies with no overlapping of confidence intervals. Additionally, I2 is large (>90%) and statistical significance for heterogeneity is considerably less than 0.05 (<0.00001).

Supplementary data 4.2: GRADE summary of findings table for obstetric and neonatal outcomes in multiparous women *(endometriosis vs. non-endometriosis).*

	Certainty assessment							№ of patients		Effect	
**Outcomes**	**№ of studies**	**Study design**	**Risk of bias (ROBINS-I)**	**Inconsistency**	**Indirectness**	**Imprecision**	**Other considerations**	**Endometriosis**	**Non-endometriosis**	**Relative (95% CI)**	**Certainty**
**Preterm Delivery**	2	observational studies[a]	serious[b]	not serious	not serious	serious[c]	none[d]	90/1271 (7.1%)	208/3821 (5.4%)	**OR 1.36** (0.97 to 1.92)	⊕⊕◯◯ Low
**Caesarean section Delivery**	2	observational studies[a]	serious[b]	not serious	not serious	serious[c]	none[d]	338/1271 (26.6%)	645/3821 (16.9%)	**OR 1.49** (0.86 to 2.57)	⊕⊕◯◯ Low
**Pre-eclampsia**	3	observational studies[a]	very serious[b]	not serious	not serious	very serious[e]	none[d]	2/179 (1.1%)	6/714 (0.8%)	**OR 1.65** (0.14 to 18.84)	⊕◯◯◯ Very low
**Hypertensive disorders of pregnancy**	3	observational studies[a]	very serious[b]	not serious	not serious	serious[c]	none[d]	63/1278 (4.9%)	182/3856 (4.7%)	**OR 1.05** (0.34 to 3.24)	⊕◯◯◯ Very low

CI: confidence interval; **OR:** odds ratio. **Explanations: a.** Includes both cohort and case-control studies; **b**. Risk of bias due to following: unrepresentativeness of either cases/exposed or controls/unexposed, not adjusting for confounders and/or small sample size; **c.** Downgraded one level for small sample size and the 95% CI overlaps no effect and is wide; **d.** There are too

few studies to accurately detect funnel plot asymmetry (<10). Therefore, the possibility of publication bias is not excluded but it is not considered sufficient to downgrade the quality of evidence; **e.** Downgraded two levels for small sample size with very small number of events and the 95% CI overlaps no effect and is wide.

The role of parity in the relationship between endometriosis and pregnancy outcomes.

Yorain Sri Ranjan, Nida Ziauddeen, Beth Stuart, Nisreen Alwan, Ying Cheong

Supplementary data 4.3: GRADE summary of findings table for obstetric and neonatal outcomes in women with endometriosis *(primiparous vs. multiparous).*

	Certainty assessment							№ of patients		Effect	
**Outcome**	**№ of studies**	**Study design**	**Risk of bias (ROBINS-I)**	**Inconsistency**	**Indirectness**	**Imprecision**	**Other considerations**	**Primiparous**	**Multiparous**	**Relative (95% CI)**	**Certainty**
**Preterm Delivery**	2	observational studies[a]	serious[b]	not serious	not serious	serious[c]	none[d]	278/3277 (8.5%)	90/1271 (7.1%)	**OR 1.49** (0.73 to 3.07)	⨁⨁◯◯ Low
**Caesarean section delivery**	2	observational studies[a]	serious[b]	not serious	not serious	serious[c]	none[d]	1042/3277 (31.8%)	338/1271 (26.6%)	**OR 1.41** (0.96 to 2.08)	⨁⨁◯◯ Low
**Gestational Hypertension**	2	observational studies[a]	very serious[b]	not serious	not serious	very serious[e]	none[d]	14/261 (5.4%)	1/104 (1.0%)	**OR 3.29** (0.59 to 18.23)	⨁◯◯◯ Very low
**Pre-eclampsia**	3	observational studies[a]	very serious[b]	not serious	not serious	very serious[e]	none[d]	15/431 (3.5%)	2/179 (1.1%)	**OR 1.88** (0.47 to 7.54)	⨁◯◯◯ Very low
**Hypertensive disorders of pregnancy**	3	observational studies[a]	serious[b]	not serious	not serious	serious[c]	none[d]	317/3319 (9.6%)	63/1278 (4.9%)	**OR 1.99** (1.50 to 2.63)	⨁⨁◯◯ Low

CI: confidence interval; **OR:** odds ratio. **Explanations: a.** Includes both cohort and case-control studies; **b.** Risk of bias due to following: unrepresentativeness of either cases/exposed or controls/unexposed, not adjusting for confounders and/or small sample size; **c.** Downgraded one level for small sample size (optimal information size criteria is not met) and/or the 95% CI overlaps no effect and is wide; **d.** There are too few studies to accurately detect funnel plot asymmetry (<10). Therefore, the possibility of publication bias is not excluded but it is not considered sufficient to downgrade the quality of evidence; **e.** Downgraded two levels for small sample size with very small number of events and the 95% CI overlaps no effect and is wide.

The role of parity in the relationship between endometriosis and pregnancy outcomes.

Yorain Sri Ranjan, Nida Ziauddeen, Beth Stuart, Nisreen Alwan, Ying Cheong
